# Secoisolariciresinol Diglucoside Improves Ovarian Reserve in Aging Mouse by Inhibiting Oxidative Stress

**DOI:** 10.3389/fmolb.2021.806412

**Published:** 2022-01-04

**Authors:** XueLai He, Yong Wang, MeiQi Wu, JiangChun Wei, XianDuo Sun, AnHua Wang, GaoSheng Hu, JingMing Jia

**Affiliations:** School of Traditional Chinese Materia Medica, Shenyang Pharmaceutical University, Shenyang, China

**Keywords:** ovarian metabolomics, ovarian reserve, ovary aging, reproductive aging, oxidative stress, reactive oxygen species, secoisolariciresinol diglucoside

## Abstract

Ovarian reserve is a key factor in the reproductive function of the ovaries. Ovarian aging is characterized by a gradual decline in the quantity and quality of follicles. The underlying mechanism of ovarian aging is complex and age-related oxidative stress is considered one of the most likely factors. Secoisolariciresinol diglucoside (SDG) has been shown to have good scavenging ability against reactive oxygen species (ROS) which slowly accumulates in ovarian tissues. However, it is unknown whether SDG had beneficial effects on aging ovaries. In this study, we used 37-week-old female C57BL/6J mouse as a natural reproductive aging model to evaluate the role of SDG in ovarian aging. SDG (7 and 70 mg/kg) intragastric administration was performed in the mice daily. After 8 weeks, the effects of SDG on aging ovaries were evaluated by counting the number of follicles and the expression of follicle-stimulating hormone receptors (FSHR) in the ovary. The mechanism of SDG on the aging ovaries was further explored through ovarian metabolomics. It was found that SDG can effectively increase the number of growing follicles and increase the expression of the FSHR protein. The metabolomics results showed that the ovaries in the SDG intervention group achieved better uptake and transport of nutrients, including amino acids and glucose that are necessary for the development of oocytes. At the same time, the ovaries of the SDG intervention group showed that the drug reduced ROS generation. Additionally, we found that ovarian telomere length and ovarian mitochondrial DNA copy number that are highly susceptible to ROS damage and are also related to aging. The results showed that SDG can significantly increase mitochondrial DNA copy number and slow down the process of telomere shortening. These data indicate that SDG improves ovarian reserve by inhibiting oxidative stress.

## Introduction

Delayed childbearing age is an important social change. The fertility of women is adversely affected by aging which reduced ovarian reserve. Ovarian reserve can be measured by the quantity and quality of follicles (Gosden et al., 1983; Tatone et al., 2008). Although the molecular mechanism of ovarian aging is not fully understood, the negative effect of oxidative stress such as the accumulation of reactive oxygen species (ROS), is considered one of the most likely factors (Tarín, 1996; Grøndahl et al., 2010). Telomere length is an important factor in aging. There is a positive association among the telomere length of cumulus cells and the quality of oocytes and embryos (Cheng et al., 2013). Telomere shortening impairs meiosis and embryonic development (Keefe and Liu, 2009). Telomeres are highly sensitive to ROS. Mitochondria are closely related to ROS dynamics. They are essential for generating energy in newly fertilized oocytes and early embryonic development. Moreover, mitochondria are closely associated with the age-related decline in oocyte quality in both mouse models and human (Santos et al., 2006; Kushnir et al., 2012; Murakoshi et al., 2013).

An important sign of reproductive aging is an increased level of follicle-stimulating hormone (FSH) (Velde et al., 1997). FSH interacts with follicle-stimulating hormone receptors (FSHR) to ensure the development and maturation of granulosa cells and oocytes (Dias et al., 2002). FSH affects the quantity and quality of follicles. The level of FSHR in granulosa cells is critical for the development of oocytes (Cai et al., 2007).

Flaxseed has a long history of human consumption and can be added to bread, pasta, muffins, and cookies (Mercier et al., 2014). Secoisolariciresinol diglucoside (SDG) is the most important component of flaxseed in addition to flax oil. Flaxseed is a good natural antioxidant (Kitts et al., 1999; Hu et al., 2007), and regulates a variety of cell signaling pathways that affect the development of various diseases, such as menopausal discomfort, cardiovascular disease, and diabetes (Imran et al., 2015). Previous studies showed that SDG can be metabolized in mammals to enterodiol and enterolactone, and exert estrogen-like effects (Setchell et al., 2014). In addition, SDG can penetrate the blood-brain barrier and accumulate in reproductive tissues, such as the ovaries, uterus, and breasts (Saarinen and Thompson, 2010).

However, little is known on whether SDG has beneficial effects on aging ovaries. In this study, we used 37-week-old C57BL/6J mice as a natural reproductive aging model and 6-week-old mice as the controls to explore the effect of SDG on aging ovaries.

## Materials and Methods

### Animals

Female C57BL/6J mice, aged 6 and 37 weeks, were purchased from GemPharmatech Co. Ltd. and housed at Shenyang Pharmaceutical University in a specific pathogen-free experimental animal center. All procedures were performed in accordance with the guidelines of the National Institutes of Health. Mice were kept in a 12:12 h light: dark cycle with standard temperature and humidity, and free access to food and water.

**FIGURE 1:**
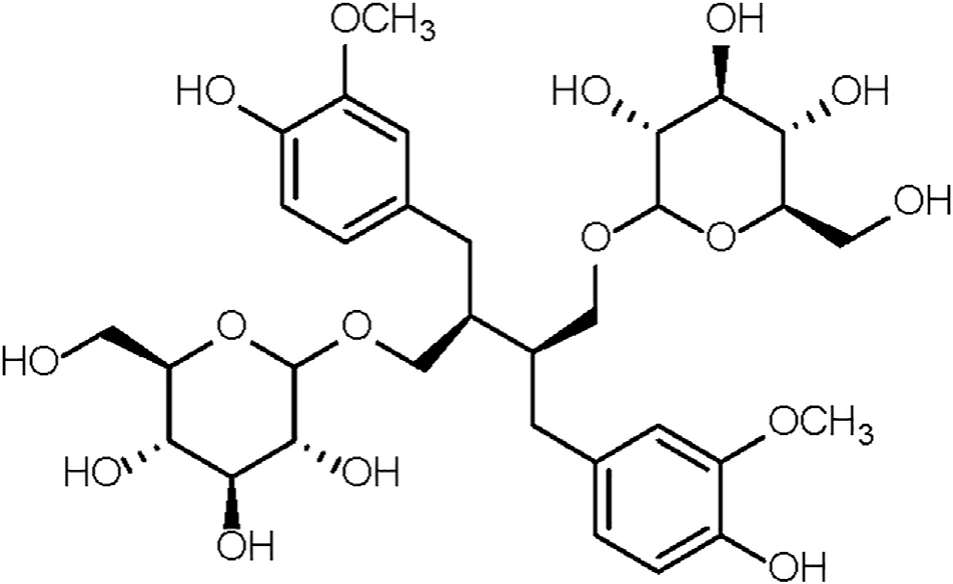
The chemical structure of SDG.

The 37-week-old mice were randomly divided into 3 groups. The first group of mice, together with 6-week-old mice, were given saline. These two groups were designated as the old control group (Old) and the young control group (Young). The second and third groups received, respectively, 7 and 70 mg/kg of SDG in saline. These two groups were designated as the low-dose SDG group (SDG-L) and the high-dose SDG group (SDG-H). A solution of SDG (Chengdu Biopurify Phytochemicals Ltd., CAS NO.158932-33-3, Purity 98%) in saline was freshly prepared daily before intragastric administration in mice. The chemical structure of SDG is shown in Figure 1. The SDG and saline were administered at the same administration time point, with the same researcher and in the same operating room. The mice were sacrificed after daily treatment for 8 weeks. The ovaries were dissected and immediately fixed in 4% tissue cell fixation solution (Beijing Dingguo Changsheng Biotechnology Co. Ltd.). Parts of the ovaries were rinsed in saline, rapidly frozen in liquid nitrogen, and stored at −72°C until processing.

There was no exclusion during the experiment and statistical analysis. The follicle counting and evaluation of the ovary immunofluorescence were blinded. Throughout the experiment, animal care staff were unaware of allocation groups to ensure that all animals in the experiment were handled, monitored, and treated in the same way (Percie du Sert et al., 2020).

### Serial Sectioning of the Ovary and Counting of the Follicles

After immersion in 4% tissue cell fixation solution for over 24 h, tissues from 5 randomly selected mice were embedded in paraffin. According to previous methods (Bolon et al., 1997), serial sections (5 µm) were cut from each ovary and were then aligned in order on glass microscopic slides. Each fifth section was stained with hematoxylin and eosin. All stained sections were analyzed after scanning with a Pannoramic DESK (3D HISTECH Ltd.) following the manufacturer’s instructions. The total number of follicles per whole ovary was calculated by combining all the counts.

The follicles were classified as primordial and primary (an oocyte surrounded by a single layer of squamous or cuboidal granulosa cells), secondary (having more than one layer of surrounding granulosa cells and with no visible antrum), or antral (possessing an area of follicular fluid or antral space) follicles according to a previous study (Myers et al., 2004). Based on the different sizes of the follicles, every 5th section was analyzed for primordial and primary follicles, every 20th section was analyzed for secondary follicles, and every 40th section was analyzed for antral follicles (Tamura et al., 2017).

### Ovary Immunofluorescence Staining

Three paraffin sections in the middle part of ovaries per mouse and 3 mice each group were dewaxed, and the antigens were heat-recovered in EDTA antigen retrieval buffer (pH 8.0, Wuhan Servicebio Technology Co. Ltd.). Sections were blocked with BSA (Wuhan Servicebio Technology Co. Ltd.) for 30 min, then incubated with rabbit anti-FSHR antibody (1:200) (Wuhan Servicebio Technology Co. Ltd.) at 4°C overnight, followed by biotinylated Cy3 goat anti-rabbit secondary antibody (Wuhan Servicebio Technology Co. Ltd.) for 50 min at room temperature in the dark. DAPI dye (Wuhan Servicebio Technology Co. Ltd.) was added and incubated for another 10 min to stain the nuclei. The sections were then incubated in autofluorescence quencher for 5 min. Slice observation and image capture were performed with a fluorescence microscope (NIKON ECLIPSE C1.) and an imaging system (NIKON DS-U3). Intensity analysis was applied to measure the expression of FSHR protein.

### Ovarian Metabolomics Analysis

At the end of the 8-weeks SDG intervention, ovarian samples from 36 mice each group were collected for metabolomic analysis. A combination of an UPLC-MS/MS detection platform and multivariate statistical analysis was used to study the metabolome differences between samples (Chen et al., 2013). The identification and quantification of the metabolites were performed at Wuhan MetWare Biotechnology Co. Ltd. Methods of sample preparation and extraction, and UPLC-MS/MS conditions are described in the Supplementary Material. A VIP (variable importance in projection) value of not less than 1.0 and a fold change of not less than 1.4 for each metabolite were used as combined cut-off points for statistical significance. The differential metabolites were annotated and further analyzed by Ingenuity Pathway Analysis (IPA) (Krämer et al., 2014). Disease or Functions Analysis was performed to predict related functions among the differential metabolites by IPA.

### Measurement of Telomere Length by Quantitative Real-Time PCR

The average telomere length was measured using a real-time PCR assay as previously described (Gil and Coetzer, 2004). Briefly, telomere length was quantified by the ratio of the amount of the telomere amplification product (T) to the amount of product from a single-copy gene (S). The single-copy gene *36B4* was used as the control. The PCR reaction was performed on a CFX96 real-time PCR system (Bio-Rad). The genomic template DNA was extracted using a Total DNA Extraction Kit (Tiangen Biotech Co. Ltd.). The primers used to amplify the telomere DNA and the control gene *36B4* were designed using Primer Premier software (Premier Biosoft Inc., CA) and are shown in Table1. The conditions for the PCR reaction were 95°C for 5 min and 40 cycles of 95°C for 10 s, 60°C for 30 s. The telomere DNA was normalized to that of *36B4* to generate a T/S ratio which indicates the relative length of the telomere.

**TABLE 1:** Primer sequences used in this research.

Genes	Primers
*TELO*	F 5′-CGG​TTT​GTT​TGG​GTT​TGG​GTT​TGG​GTT​TGG​GTT​TGG​GTT-3′
	R 5′-GGC​TTG​CCT​TAC​CCT​TAC​CCT​TAC​CCT​TAC​CCT​TAC​CCT-3′
*36B4*	F 5′-ACA​CTC​CAT​CAT​CAA​TGG​GTA​CAA-3′
	R 5′-TCA​GTA​AGT​GGG​AAG​GTG​TAC​TCA​G-3′
*Cox1*	F 5′-CAG​GAG​CAG​GAA​CAG​GAT​GA-3′
	R 5′-GCA​CCT​AAA​ATA​GAC​GAC​ACC​C-3′
*Nthl1*	F 5′-GGG​AAT​ACT​GAG​GCA​GAG​GAC-3′
	R 5′-AGG​TGA​AGA​AGG​CAG​CAA​AG-3′

### Mitochondrial DNA Copy Number Quantification

The ratio of the levels of the single-copy mitochondrial gene *Cox1* (cytochrome c oxidase) to the single-copy nuclear gene *Nthl1* (*n*th endonuclease III-like 1) was used to determine the mitochondrial DNA copy number as described in a previous study (Aiken et al., 2016). Real-time PCR was performed to obtain the average copy number of mitochondrial DNA/nuclear DNA. Total DNA was extracted using a Total DNA Extraction Kit (Tiangen Biotech Co. Ltd.), from 9 mice per group. Primer design and PCR conditions were the same as those used for telomere length measurement. The method for calculating the average mitochondrial DNA copy number followed that described in a previous study (Nicklas et al., 2004).

### Statistical Analysis

All experimental data were expressed as mean ± SEM, and subjected to one-way analysis of variance with the Tukey test or Dunnett’s T3 test where appropriate. The nonparametric Kruskal-Wallis test was performed when normality or equality of variances were not met. Statistical analysis was performed with GraphPad Prism 8.0 (GraphPad software) and a *p* value less than 0.05 was considered significant.

## Results

### SDG Increased the Number of Growing Follicles

**FIGURE 2:**
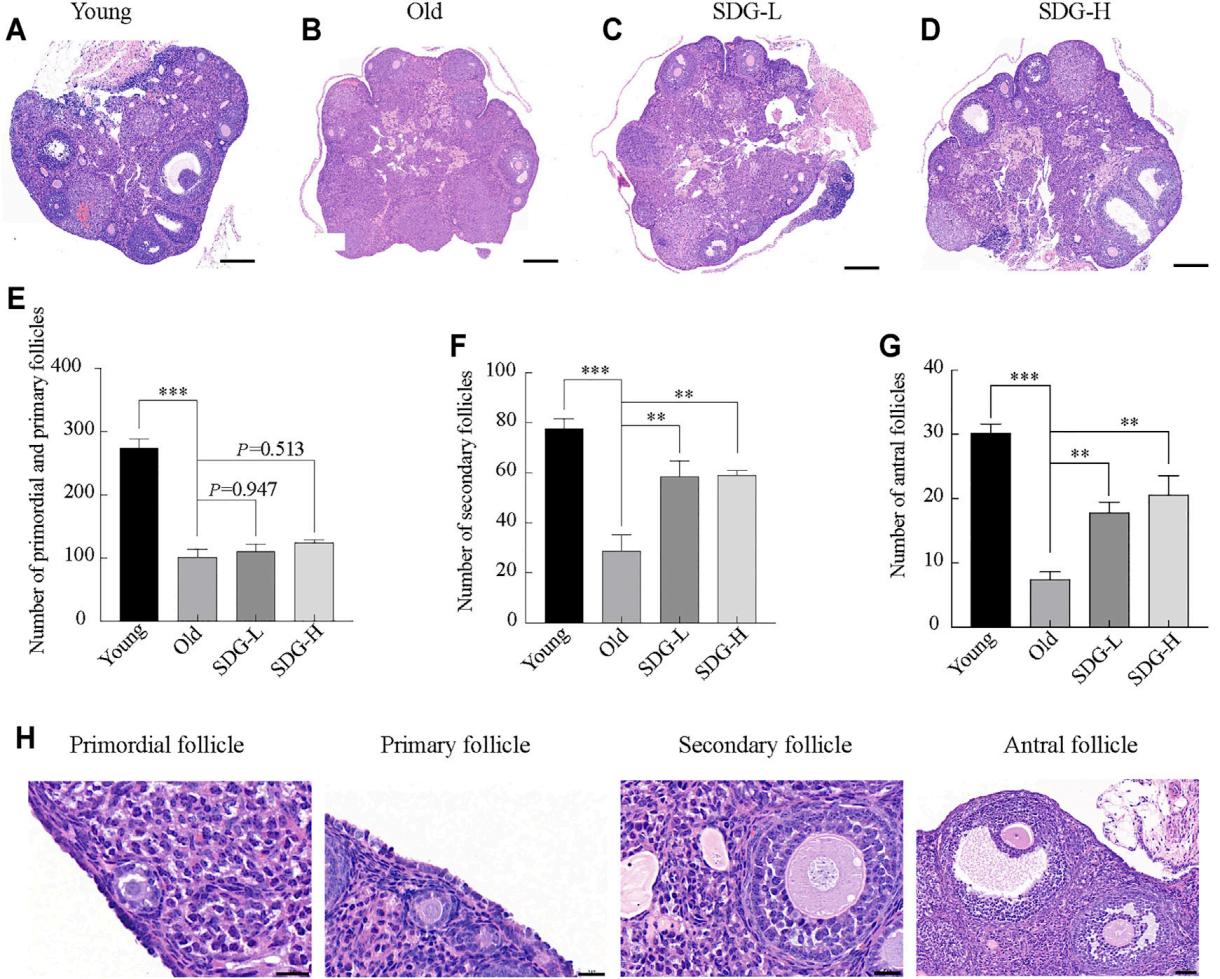
Ovary morphology and follicle counting in mice after intragastric administration of SDG. **(A–D)** Morphology of ovaries from each group of mice. Scale bar, 200 µm. Representative samples from Young **(A)** and the Old **(B)** mice treated with saline, SDG-L **(C)** and SDG-H **(D)** with a concentration of 7 and 70 mg/kg daily for 8 weeks **(E–G)** The number of primordial and primary **(E)**, secondary **(F)**, and antral **(G)** follicles. The serial sections from each ovary were stained with hematoxylin and eosin. The total number of follicles per whole ovary was calculated by combining the counts. The morphology of different types of follicles are shown in **(H)**. Primordial and primary follicles, scale bar, 20 μm; secondary follicles, scale bar, 20 μm; antral follicles, scale bar, 50 µm. The data are expressed as mean ± SEM. * *p* < 0.05, ** *p* < 0.01, *** *p* < 0.001. The *p* value is labeled if *p* > 0.05.

As expected, many follicles, including primordial, primary, secondary, and antral follicles, located in the ovarian cortex in young control mice (Figure 2A). In old control mice, the ovaries exhibited a markedly reduced number of follicles at all stages (Figure 2B). The old mice treated with SDG had significantly more secondary and antral follicles than the old controls (Figures 2F,G). However, both low and high doses of SDG did not improve the number of primordial and primary follicles (Figure 2E, *p* = 0.947 and *p* = 0.513 respectively). The morphology of different types of follicles are shown in Figure 2H.

### SDG Increased FSHR Protein Expression

**FIGURE 3:**
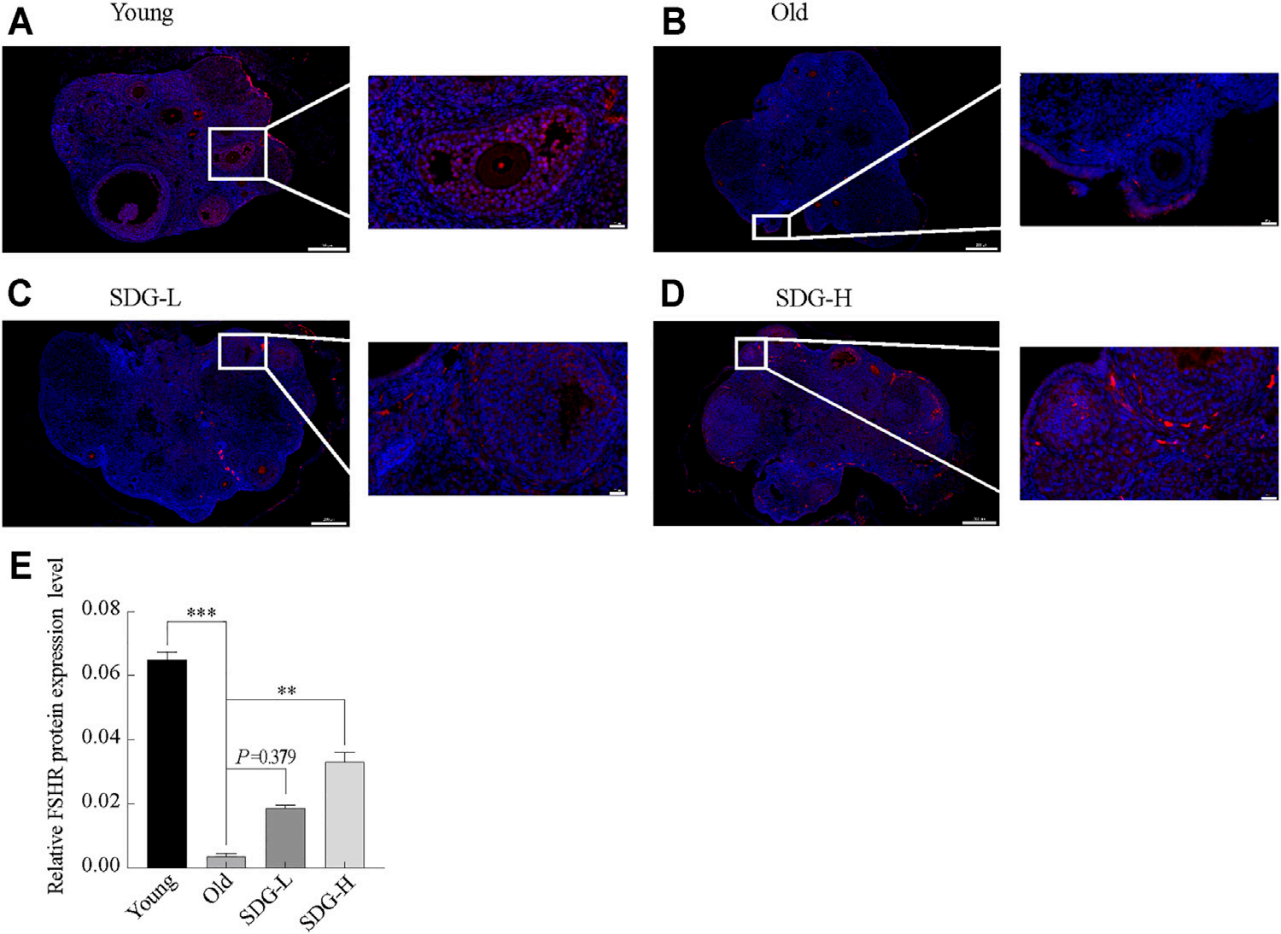
Immunofluorescence staining of FSHR protein in the ovaries of mice after intragastric administration of SDG. **(A–D)** Images of immunofluorescence staining of FSHR protein in the ovaries of each group of mice, the Young **(A)**, the Old **(B)**, the SDG-L **(C)**, and the SDG-H **(D)**. Scale bar, 200 and 20 µm respectively. FSHR protein was detected by a fluorescein-tagged antibody (Red), and nuclei were stained by DAPI dye (Blue). **(E)** The comparison of fluorescence intensity of FSHR protein in ovary sections in different groups. The data are expressed as mean ± SEM. * *p* < 0.05, ** *p* < 0.01, *** *p* < 0.001. The *p* value is labeled if *p* > 0.05.

The location of FSHR protein in granulosa cells is shown in Figures 3A–D. FSHR was detected by a fluorescein-tagged antibody, and appears red. DAPI-stained nuclei appear blue. The fluorescence intensity analysis showed that the expression of FSHR in the old control group was significantly reduced compared to the young control group (Figure 3E). Marked red fluorescence enhancement was observed in the SDG-H group compared to the old control group. This result indicated that high-dose SDG induced the expression of FSHR. However, the effect of low-dose SDG was not significant (Figure 3E, *p* = 0.379).

### Ovary Metabolomics Analysis

**FIGURE 4:**
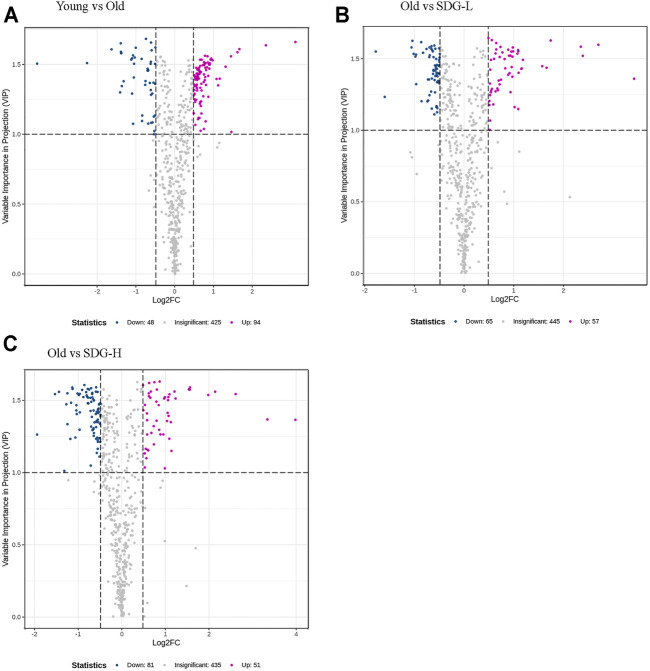
Filtered differential metabolites in the ovaries of mice in different groups. The comparisons of the Young and the Old **(A)**, the Old and the SDG-L **(B)** and the Old and the SDG-H **(C)** were analyzed. Each point in the volcano map represents a metabolite, the abscissa represents the logarithm of the quantitative difference of a certain metabolite in the two samples; the ordinate represents the VIP value. The larger the absolute value of the abscissa, the greater the difference between the two samples; the larger the ordinate value, the more significant and the more reliable the differential levels of metabolites. The blue dots in the figure represent downregulated differential metabolites, the magenta dots represent upregulated differential metabolites, and the gray dots represent the metabolites that are detected but not significantly different.

A total of 567 metabolites were detected by the widely targeted metabolomics analysis. Limiting the VIP value to not less than 1.0 and the fold change between different groups to not less than 1.4, we obtained 142 metabolites from the comparison of the old control group and the young control group. 122 and 132 metabolites were obtained from comparisons between the old control group and the SDG-L group and the SDG-H group, respectively. These filtered differential metabolites are shown in the volcano plot (Figure 4).

### Ingenuity Pathway Analysis

**FIGURE 5:**
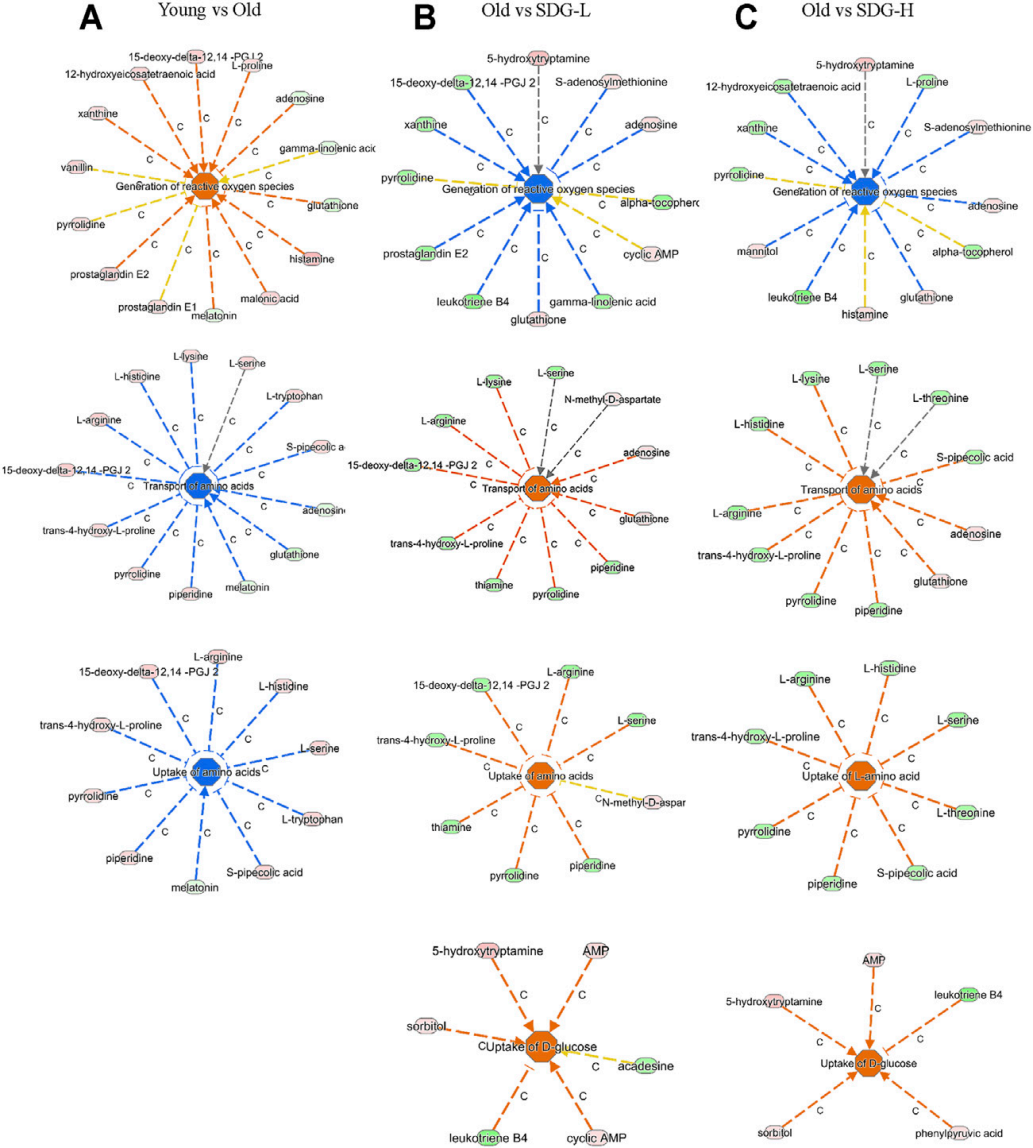
The related differential metabolites in each Disease or Functions Annotation by IPA in different groups. The comparisons of the Young and the Old **(A)**, the Old and the SDG-L **(B)**, and the Old and the SDG-H **(C)** are shown. The disease states and biological functions are in the center; blue represents a decrease and orange represents an increase. The metabolites in light green represent a quantitative decrease, while in pink represent an increase. The lines between the annotation and the metabolites represent the predicted relationship. The line in blue leads to inhibition, the line in orange leads to activation, the line in yellow represents the findings is inconsistent with the state of downstream molecule, and the line in grey represents the effect not predicted.

Ingenuity Pathway Analysis (IPA) uses the activation z-score algorithm to make predictions. A positive value indicates an increase, a negative number represents a decrease, and a higher absolute value of the z-score indicates a greater degree of change between groups. We used Disease or Function analysis to associate molecules to known disease states and biological functions. As shown in Table 2, the differential metabolites in the old controls and young controls are related to an increased generation of reactive oxygen species (ROS) with a positive value of the z-score, and the decreased transport of amino acids and uptake of amino acids with negative values of the z-score. These scores were reversed by SDG intervention. In addition, we found that SDG participates in carbohydrate metabolism and improves glucose uptake. The related differential metabolites in each Disease or Functions Annotation in different comparison groups are shown in Figure 5.

**TABLE 2:** Four major disease or functions annotation identified by IPA.

Disease or functions annotation	Young vs. old		Old vs. SDG-L		Old vs. SDG-H	
	Activation z-score	*p* -value	Activation z-score	*p* -value	Activation z-score	*p*-value
Generation of reactive oxygen species	2.254	5.03E-09	−2.352	2.08E-07	−2.352	5.63E-07
Transport of amino acids	−3.075	8.44E-10	2.577	9.63E-07	2.577	1.77E-07
Uptake of amino acids	−2.765	4.71E-08	2.2	0.000102	2.433	7.5E-07
Uptake of d-glucose	—	—	2.2	0.0044	2.2	0.00682

### SDG Prevents Telomere Shortening and Reduction of Mitochondrial DNA Copy Number

**FIGURE 6:**
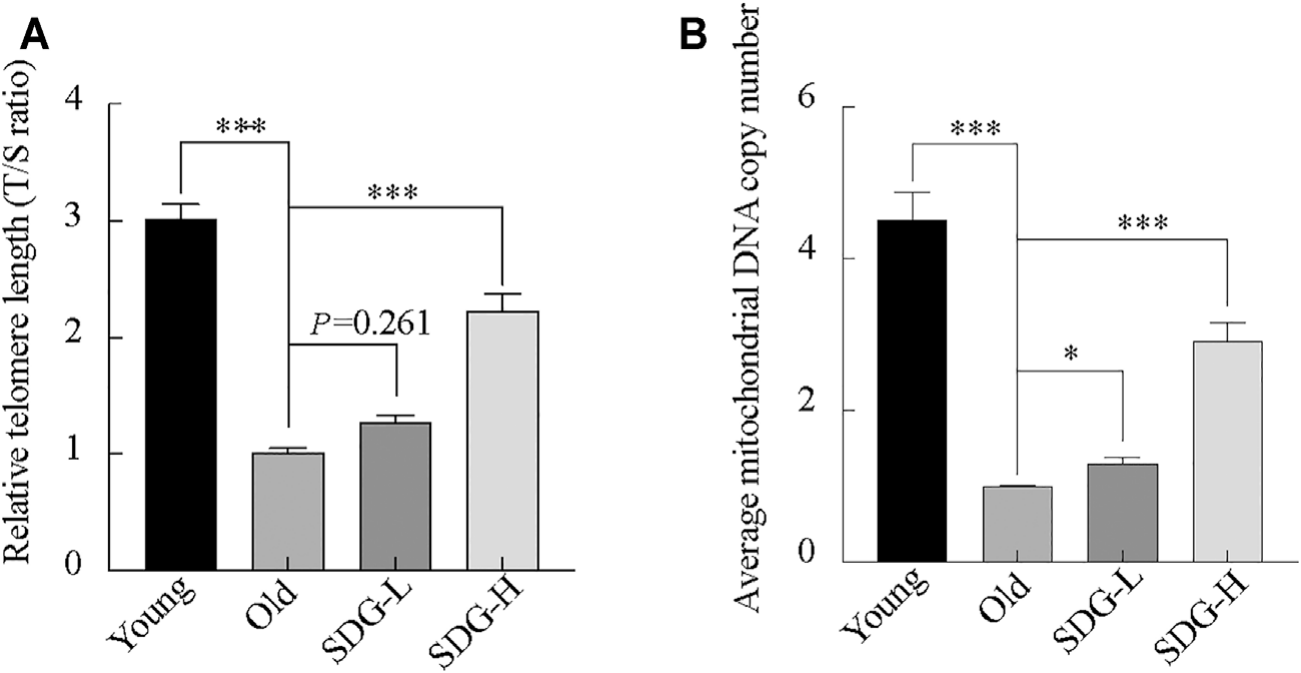
Telomere length, mitochondrial DNA copy number in the ovaries of mice after intragastric administration of SDG. **(A)** Relative telomere length was estimated as the T/S ratio by quantitative real-time PCR analysis. **(B)** Average mitochondrial DNA copy number was determined by the ratio of the levels of the single-copy mitochondrial gene to the single-copy nuclear gene by real-time PCR analysis. The data are expressed as mean ± SEM. * *p* < 0.05, ** *p* < 0.01, *** *p* < 0.001. The *p* value is labeled if *p* > 0.05.

Telomere length, estimated by the T/S ratio, showed a significant reduction in old control mice compared with young controls (Figure 6A). The old mice treated with high-dose SDG had significantly longer telomere length than the old controls. However, the effect of low-dose SDG was not significant, *p* = 0.261.

Similar to the telomere length, the mitochondrial DNA copy number in the old controls was markedly decreased compared to young controls. Both the high-dose and low-dose SDG-treated groups showed a significant increase in mitochondrial DNA copy number compared to the old control group (Figure 6B).

## Discussion

Aging has an immeasurable impact on female reproductive function (Broekmans et al., 2009; Nelson et al., 2013). Postponement of parenthood and increased maternal age have led more people to seek healthy nutritional supplements or anti-aging foods (Moslehi et al., 2017) in the hope of improving ovarian function and delaying aging. These foods/supplements include soy products (Cederroth et al., 2012), resveratrol (Cabello et al., 2015), melatonin (Song et al., 2016), and SDG. There are growing evidence showing that SDG-enriched flaxseed products offer health benefits. But the dosage of SDG associated with the outcomes of studies were complicated. Brevail of Barlean’s company, contains 50 mg SDG and is used daily for relieving symptoms of menopause, maintaining healthy breast tissue and estrogen balance. However, Adolphe et al. suggested that a dose of at least 500 mg SDG per day is needed to have significant benefits and that this dosage is safe for most people (Adolphe et al., 2010). Here, we used both doses in the study. The results showed that there was little difference between the two dosages, and the low dosage of SDG cannot improve the relative FSHR expression level and prevent telomere shortening effectively, but it can improve the ovarian reserve and decrease the oxidative stress as the high dosage of SDG.

FSHR is located exclusively in granulosa cells. This can be clearly seen in the images of the immunofluorescence staining of FSHR in the ovaries. Our results showed that the expression of the FSHR protein, as well as in the number of secondary and antral follicles, was significantly higher in the mice of young control group than in the old control group. SDG administration improved both the expression of FSHR and the number of growing follicles. These data were in consistent with previous studies, the ovarian response to FSH affects follicle development and estrogen secretion (Simoni et al., 1997). The improved expression of FSHR contributed to the hormonal balance and counteracted the discomforts of menopause resulting from the elevated levels of FSH in the blood, such as flushing (Mitchell and Woods, 2015). A high dose of SDG could be used to prevent or treat some menopausal discomforts.

It was known that, the body’s metabolic capacity and level slowly decline with age, and so will those of the ovaries. The nutritional uptake and metabolism affect ovarian function (Seli et al., 2014). In the ovaries, the significance of amino acid uptake, transport, and metabolism for oocyte development is extraordinary (Hemmings et al., 2012). In addition to the amino acids, glucose also plays an important role during follicle development (Sutton-McDowall et al., 2010). The results of Disease or Functions Annotation by IPA analysis revealed the biological functions of Transport of amino acids and Uptake of amino acids were decreased with aging (i.e., comparing old controls to young controls) and increased by SDG treatment. These data provide additional evidence supporting the beneficial role of SDG for aging ovaries and improving the follicle development.

The oxidative free radical theory of aging has been around since the 1960s (Harman, 1956). We now know that oxidative damage accumulates with aging (Davalli et al., 2016). Previous studies have reported that age-related oxidative damage occurs in interstitial cells and ovarian tissues and increases with aging (Lim and Luderer, 2011). ROS plays a vital role in aging (Harman, 1956; Harman, 1972). The main source of ROS in cells is the mitochondria. The paradox is that mitochondrial DNA lacks protection from histones or DNA binding proteins and is particularly vulnerable to ROS-mediated damage (Caston and Demple, 2017). Thus, a vicious cycle arises between mitochondrial dysfunction and ROS production, at the same time, the oxidative stress caused by the increased production of ROS due to mitochondrial respiratory dysfunction also has a significant impact on follicle formation and development (Miquel et al., 1980). We found increased level of ROS and, to some extent, dysfunctional mitochondria in the aging ovaries. In our results of Disease or Functions Annotation for the differential metabolites, the mice in the old control group showed an increase in the generation of ROS and a decrease of mitochondrial DNA copy number compared to that of the young control group. We found that SDG reduced ROS generation and increased mitochondrial DNA copy number. These data are consistent with the effects of SDG and its strong antioxidant activity (Rajesha et al., 2006; Soleymani et al., 2020). Therefore, we believe that SDG acts as an antioxidant to protect the aging ovaries.

Telomere is closely associated with aging which has been verified by previous studies. Telomere shortening is associated with reproductive senescence in mouse oocytes (Yamada-Fukunaga et al., 2013), and they are also vulnerable to oxidative damage (Smith, 2018; Coluzzi et al., 2019). Furthermore, oxidative stress and telomere shortening are exponentially related to somatic cell aging (Richter and Zglinicki, 2007). Our results showed a shorter telomere length in aging ovaries and an increased generation of ROS levels in the metabolism analysis results, suggesting there may be a relationship between these effects. We show that SDG (70 mg/kg) can prevent telomere shortening.

In this study, we report that SDG can improve ovarian reserve in the aging mouse model and is associated with oxidative stress. After 8 weeks of SDG intervention, the number of secondary and antral follicles increased, the expression level of FSHR improved, and the nutrition and energy metabolism increased in the ovaries of old mice compared to untreated old controls. In addition, SDG prevents telomere shortening and reduction in mitochondrial DNA copy number, and it decreased the generation of ROS, as evidenced from the metabolism results. Our findings contribute to the understanding that compounds with good free radical scavenging ability accumulate in the ovarian tissue, and may have the potential to benefit the ovary reserve. These findings will provide new insight and stimulate further research into additional candidate agents to combat ovarian aging.

## Data Availability

The original contributions presented in the study are included in the article/Supplementary Material, further inquiries can be directed to the corresponding author.
