# Supplementary material for: Secoisolariciresinol Diglucoside Improves Ovarian Reserve in Aging Mouse by Inhibiting Oxidative Stress
Source: Front Mol Biosci. 2022 Jan 4;8:806412. doi: 10.3389/fmolb.2021.806412 (PMC8764264; doi:10.3389/fmolb.2021.806412)
Supplement: Supplementary file 1 [file Table1.DOCX]

Supplementary Material

# Supplementary method of ovarian metabolomics

Sample preparation and extraction

The sample was thawed on ice. Take 50 mg of one sample and homogenize it with 1000 µl of ice-cold methanol/water (70%, v/v). Add cold steel balls to the mixture and homogenate for at 30 Hz for 3 min. Whirl the mixture for 1 min, and then centrifuge it with 12,000 rpm at 4℃ for 10 min. The collected supernatant will be used for LC-MS/MS analysis.

HPLC conditions

The sample extracts were analyzed using an LC-ESI-MS/MS system (UPLC, Shim-pack UFLC SHIMADZU CBM A system; MS, QTRAP® System). The analytical conditions were as follows, UPLC: column, Waters ACQUITY UPLC HSS T3 C18 (1.8 µm, 2.1 mm*100 mm); column temperature, 40℃; flow rate, 0.4 mL/min; injection volume, 2μL; solvent system, water (0.04% acetic acid): acetonitrile (0.04% acetic acid); gradient program, 95:5 V/V at 0 min, 5:95 V/V at 11.0 min, 5:95 V/V at 12.0 min, 95:5 V/V at 12.1 min, 95:5 V/V at 14.0 min.

ESI-QTRAP-MS/MS conditions

LIT and triple quadrupole (QQQ) scans were acquired on a triple quadrupole-linear ion trap mass spectrometer (QTRAP), QTRAP® LC-MS/MS System, equipped with an ESI Turbo Ion-Spray interface, operating in positive and negative ion mode and controlled by Analyst 1.6.3 software (Sciex). The ESI source operation parameters were as follows: source temperature 500℃; ion spray voltage (IS) 5500 V (positive), -4500 V (negative); ion source gas I (GSI), gas II (GSII), curtain gas (CUR) was set at 55, 60, and 25.0 psi, respectively; the collision gas (CAD) was high. Instrument tuning and mass calibration were performed with 10 and 100 μmol/L polypropylene glycol solutions in QQQ and LIT modes, respectively. A specific set of MRM transitions was monitored for each period, according to the metabolites eluted within this period.
