# Supplementary figures and images for: Secoisolariciresinol Diglucoside Improves Ovarian Reserve in Aging Mouse by Inhibiting Oxidative Stress
Source: Front Mol Biosci. 2022 Jan 4;8:806412. doi: 10.3389/fmolb.2021.806412 (PMC8764264; doi:10.3389/fmolb.2021.806412)

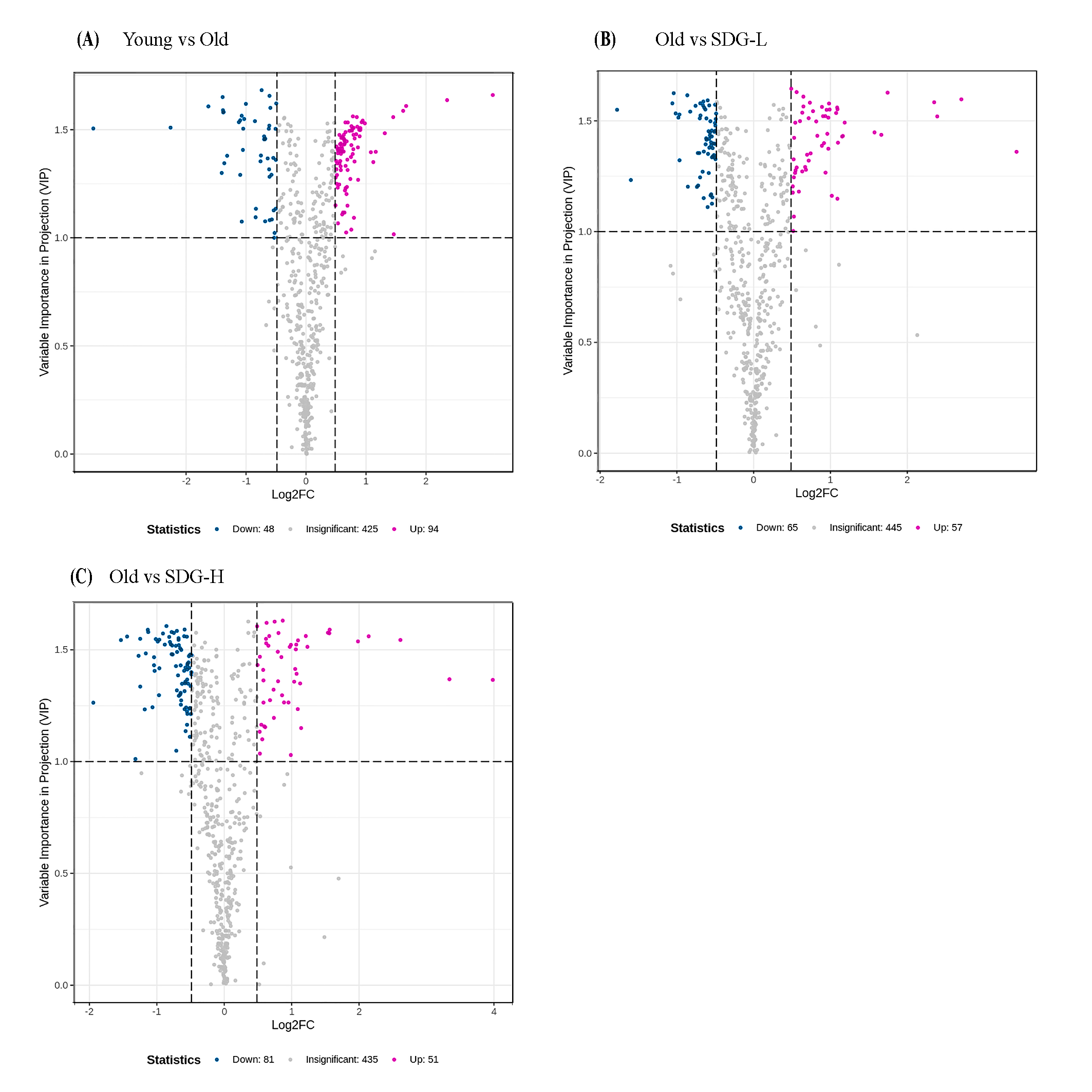

Supplement: Supplementary file 2 [file DataSheet1.ZIP › Figures/Figure 4.tiff]

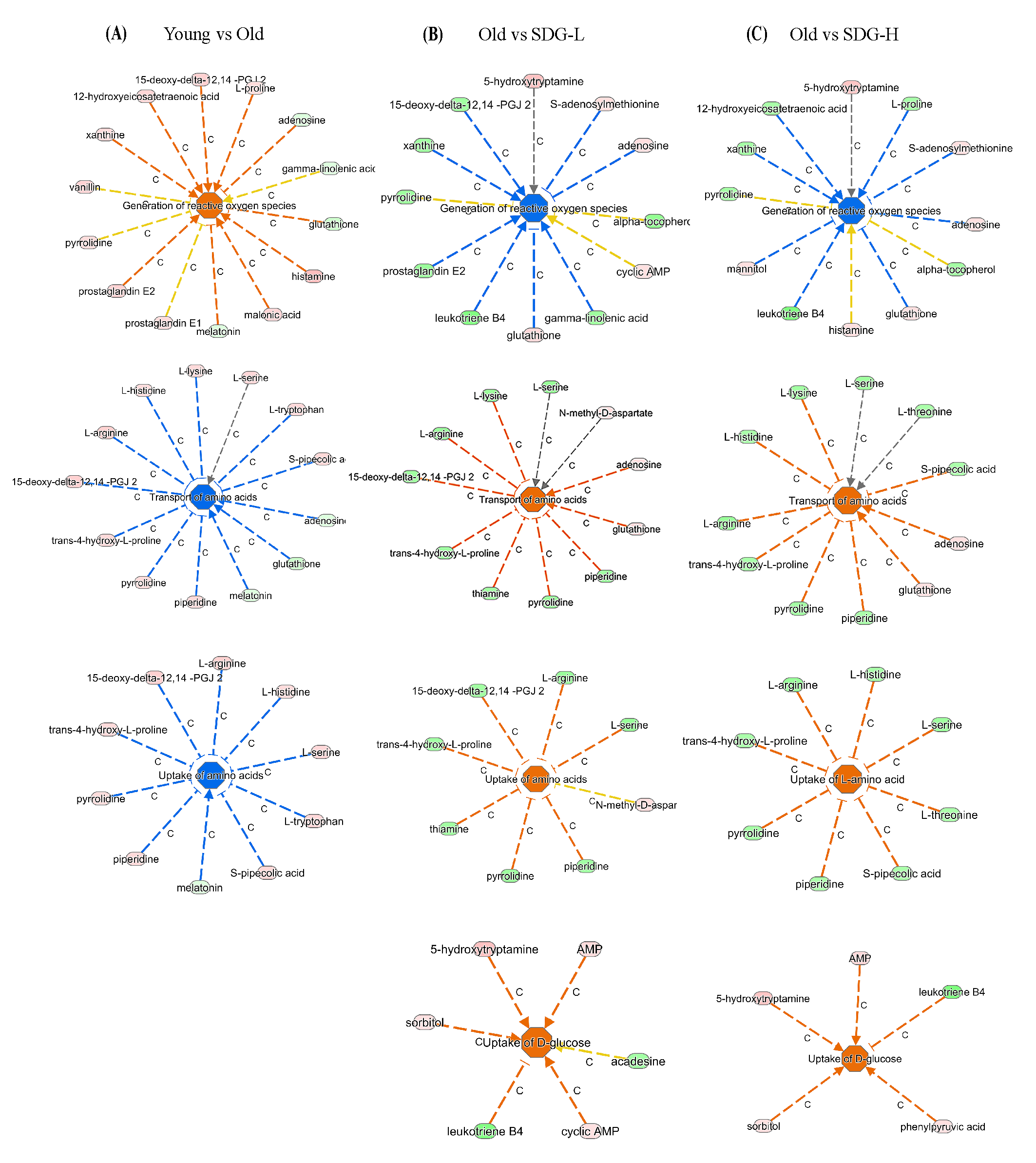

Supplement: Supplementary file 2 [file DataSheet1.ZIP › Figures/Figure 5.tiff]

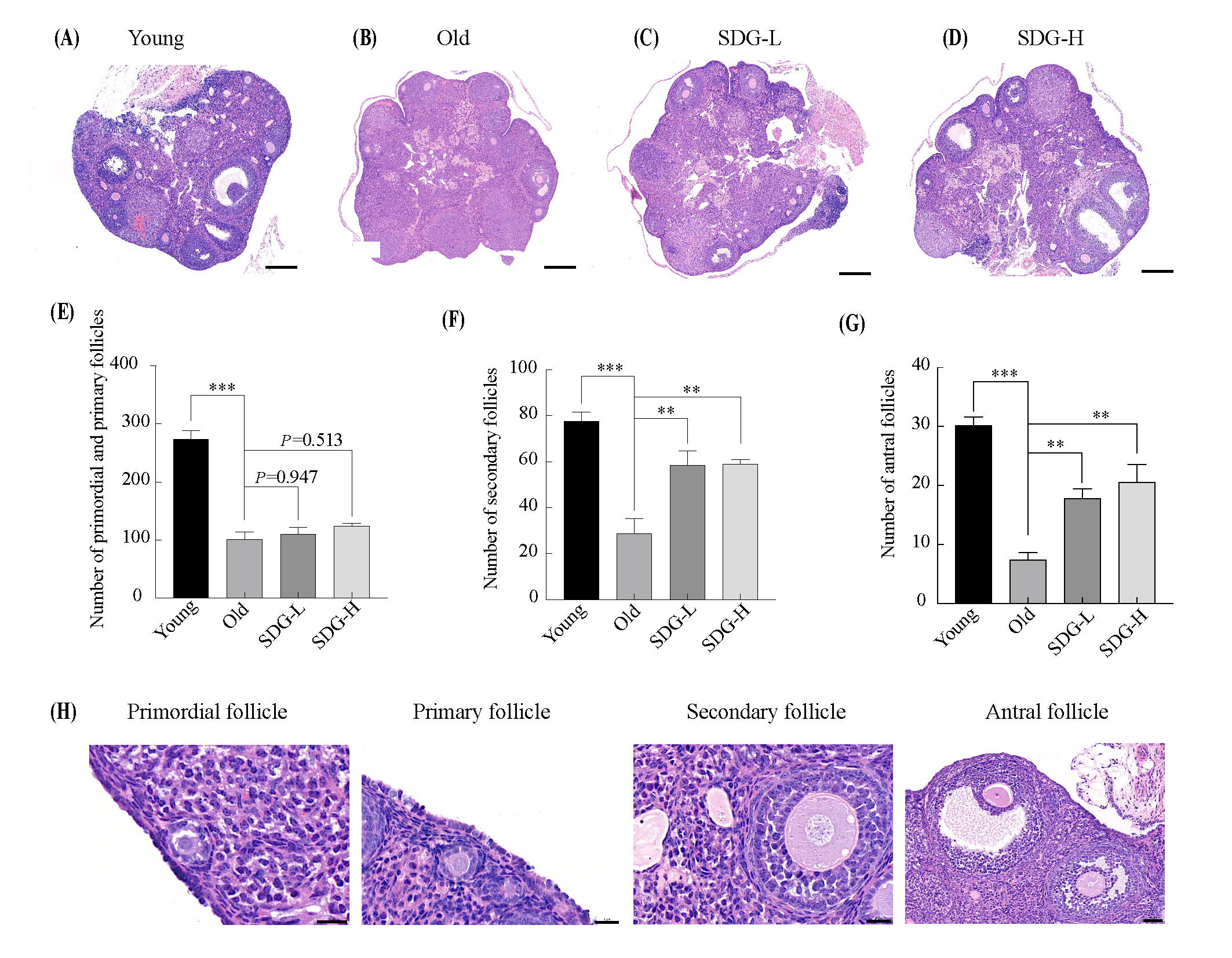

Supplement: Supplementary file 2 [file DataSheet1.ZIP › Figures/Figure 2.tiff]

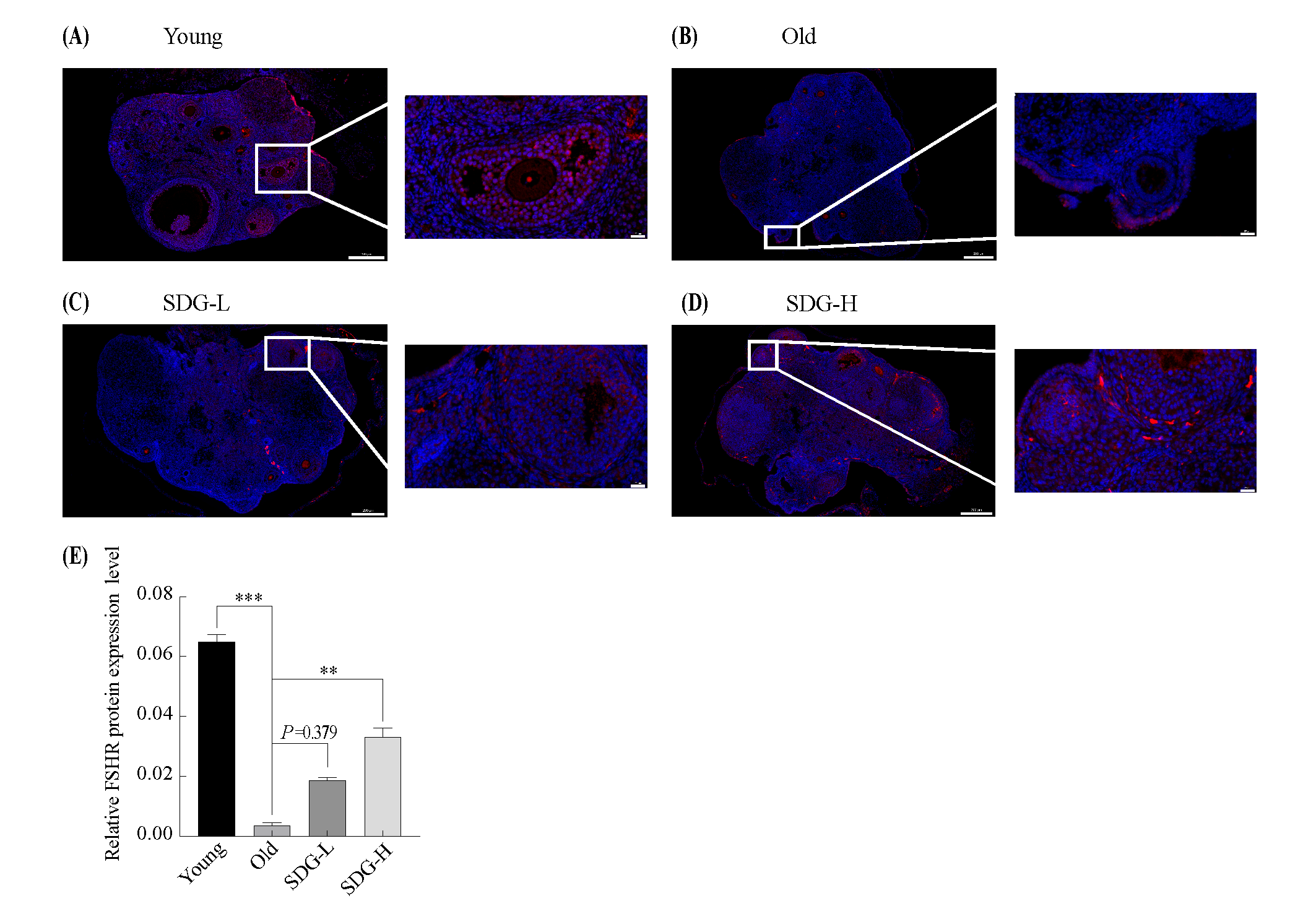

Supplement: Supplementary file 2 [file DataSheet1.ZIP › Figures/Figure 3.tiff]

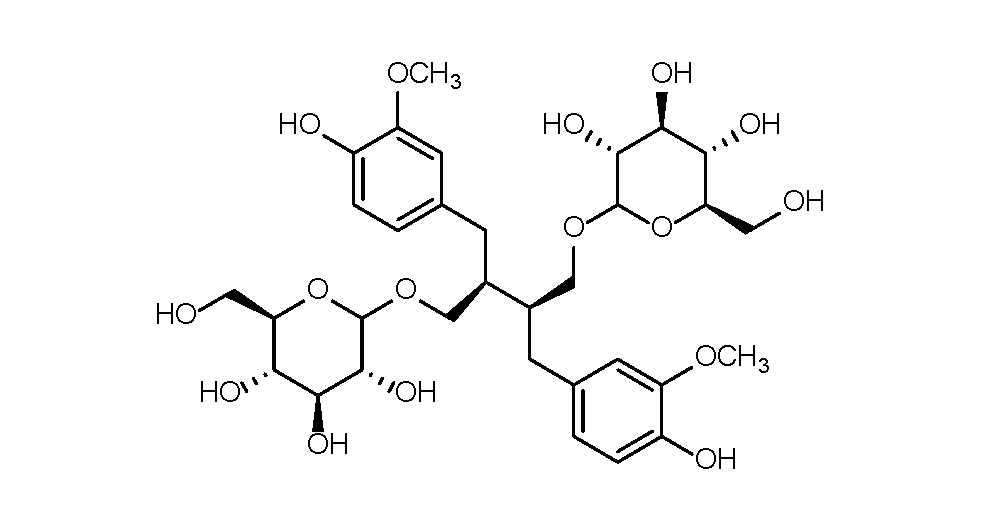

Supplement: Supplementary file 2 [file DataSheet1.ZIP › Figures/Figure 1.tiff]

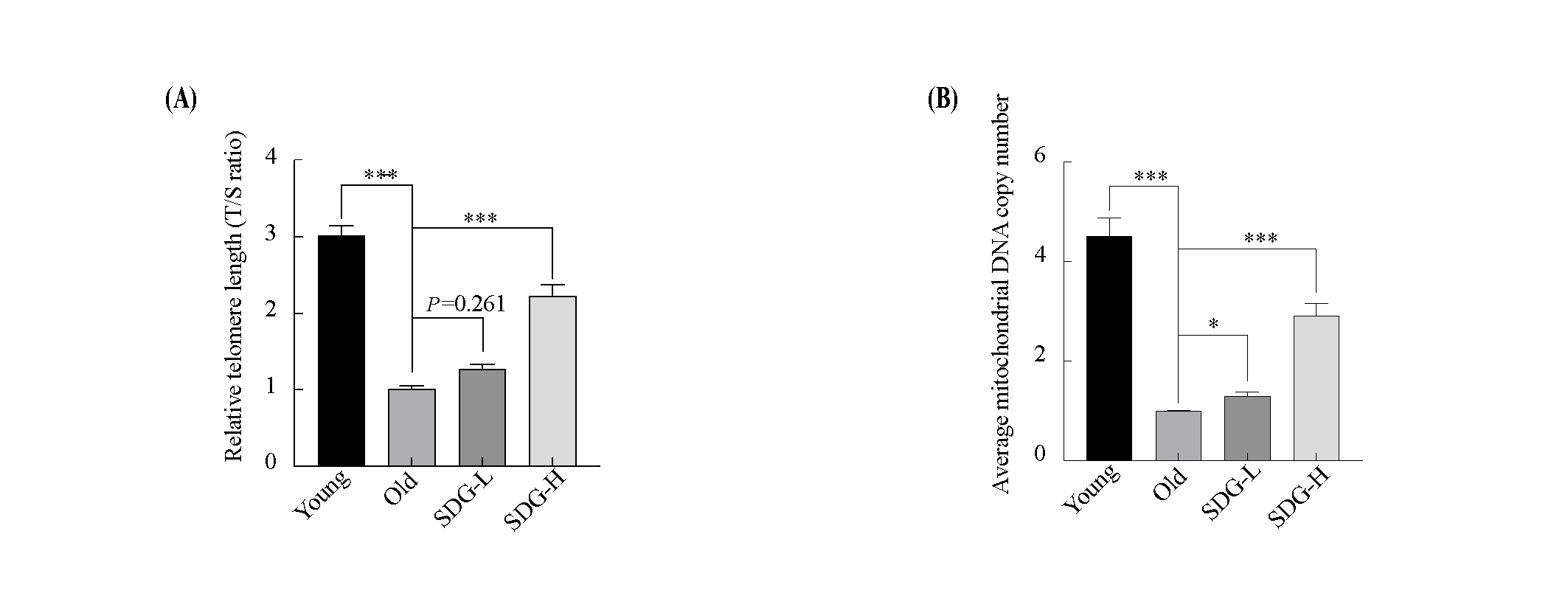

Supplement: Supplementary file 2 [file DataSheet1.ZIP › Figures/Figure 6.tiff]
